# Design and Reprogrammability of Zero Modes in 2D Materials from a Single Element

**DOI:** 10.1002/advs.202511227

**Published:** 2025-08-20

**Authors:** Daniel Revier, Molly Carton, Jeffrey I. Lipton

**Affiliations:** ^1^ Paul G. Allen School of Computer Science and Engineering University of Washington Seattle WA 98195 USA; ^2^ Mechanical Engineering University of Maryland College Park MD 20742 USA; ^3^ Mechanical and Industrial Engineering Northeastern University Boston MA 02115 USA

**Keywords:** chiral, metamaterials, mechanisms, reprogrammable, symmetry

## Abstract

Mechanical extremal materials, a class of metamaterials that exist at the bounds of elastic theory, possess the extraordinary capability to engineer any desired elastic behavior by harnessing mechanical zero modes — deformation modes that demand minimal or, ideally, no elastic energy. However, the potential for arbitrary construction and reprogramming of metamaterials remains largely unrealized, primarily due to significant challenges in qualitatively transforming zero modes within the confines of existing metamaterial design frameworks. This work presents a method for explicitly defining and in situ reprogramming zero modes of 2D extremal materials by employing straight‐line mechanisms (SLMs) and planar symmetry, which prescribe and coordinate the zero modes, respectively. The method is used to design, test, and reprogram centimeter‐scale isotropic, orthotropic, and chiral extremal materials by reorienting the SLMs in place, enabling these materials to smoothly and reversibly interpolate between extremal modalities (e.g., unimode to bimode), material properties (e.g., negative to positive Poisson's ratios), and selectively enable chirality without changing the metamaterial's global structure. This methodology provides a straightforward and explicit strategy for the design and tuning of all varieties of 2D extremal materials, enabling dynamic mechanical metamaterial construction to completely cover the gamut of elastic properties.

## Graphical abstract

Straight‐line mechanisms embedded in symmetric lattices explicitly define and reprogram zero modes in 2D extremal metamaterials. Rotating each unit cell in situ tunes extremal behaviors, spanning isotropic, orthotropic, and chiral responses. Experiments and numerical methods show continuous control of Poisson’s ratios, Young's moduli, and normal‐shear coupling without altering topology, enabling spatially programmable, adaptive extremal mechanical metamaterials.

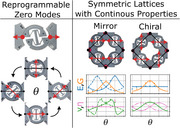

## 1 Introduction

*Mechanical extremal materials* operate at the theoretical limits of linear elasticity and are characterized by the number and type of *zero modes*, i.e., deformation modes that cost little or, ideally, no elastic energy.^[^ ^1^, ^2^, ^3^ ^]^ These materials have been developed for applications that include mechanical cloaking,^[^ ^4^, ^5^, ^6^ ^]^ acoustic cloaking,^[^ ^7^, ^8^ ^]^ and out‐of‐plane shear wave polarizers.^[^ ^9^ ^]^ Significantly, by coordinating and composing zero material modes, we can theoretically construct any feasible linear elastic material behavior.^[^ ^1^ ^]^ Although recent advances have brought reprogrammability to extremal materials, allowing them to switch between a preset number and type of zero modes,^[^ ^3^ ^]^ the designs are highly specialized, and their zero modes cannot be arbitrarily constructed. Crucially, there has not been a demonstration of a single framework to produce a variety of zero modes with different material symmetries, e.g., chiral, orthotropic, or isotropic. Without a structured design framework, previous work has resorted to application‐specific designs of metamaterials using human effort or costly computational techniques.^[^ ^10^, ^11^, ^12^, ^13^, ^14^, ^15^, ^16^, ^17^, ^18^, ^19^, ^20^, ^21^ ^]^ Thus, a framework that is able to explicitly define, tune, and coordinate the zero modes of a material is a far more comprehensive method to create extremal behavior.

Here we show an expressive and reprogrammable method to directly engineer zero modes for 2D extremal materials using compliant straight‐line mechanisms (SLMs) and planar symmetry. We base our design on the SLM because it is inherently extremal. The SLM constrains motion to a single straight‐line trajectory,^[^ ^22^, ^23^, ^24^, ^25^, ^26^ ^]^ and thus operates as an explicitly definable zero mode unit cell. We also design our SLMs to be innately reprogrammable through a rotationally symmetric construction, allowing them to pivot in place and modify the zero modes of the metamaterial without altering the global structure. To realize the SLMs as a comprehensive metamaterial, the global construction and coordination of the zero modes is performed using planar symmetry which integrates multiple SLMs onto 2D lattices to achieve the desired extremal behaviors.

This approach achieves centimeter‐scale extremal materials of various material symmetries — isotropic, orthotropic, and chiral. It also includes the ability to reprogram these materials in situ to smoothly and reversibly interpolate between different extremal modes (e.g., unimode to bimode) and emergent properties (e.g., negative to positive Poisson's ratio). Unlike other methods, our framework enables rapid, arbitrary, continuous, and spatially independent adjustment of engineered zero modes. Thus, we are able to uniquely realize Milton and Cherkaev's concept of 2D extremal material lamination^[^ ^1^ ^]^ and facilitate dynamic adjustments of material properties on demand without costly computational methods or lengthy reprogramming procedures. We design, simulate, fabricate, and validate the SLMs and symmetry as a programmable and tunable framework to define the zero modes of extremal materials, demonstrating how SLM metamaterials can access all possible 2D mechanical extremal modes. By examining both the engineering constants (Young's moduli (*E*_1_, *E*_2_), shear modulus (*G*_12_), Poisson's ratios (ν_12_, ν_21_), the normal‐shear coupling ratio (η_121_, η_122_), and the zero modes of the extremal materials' homogenized elastic matrix *C*, we discover that the orientation of the SLM and the employed symmetry pattern comprehensively determine the emergent properties and extremal behavior.

The core contributions of this paper include: 1) the use of SLMs as continuously reprogrammable zero‐mode metamaterial cells, 2) the use of symmetry to coordinate zero modes and realize extremal types of varied material symmetries (isotropic, orthotropic, chiral), and 3) a demonstration of smoothly reprogramming extremal mode and emergent properties of our extremal materials. Leveraging these insights and contributions, we extend and realize the design space of reprogrammable 2D extremal metamaterials and demonstrate their temporal and spatial variability.

## 2 Innovation and Methodology

### 2.1 SLMs are Reprogrammable Unimode Unit Cells

**Figure 1:**
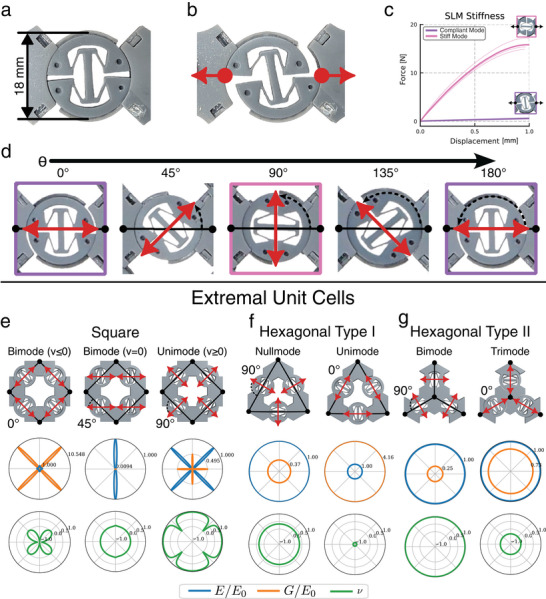
The SLM enables reprogrammable material properties by modifying the zero modes of extremal materials. The SLM is shown at rest a) and under horizontal loading b) showing linear displacement along the straight‐line trajectory when loaded horizontally. c) A single fabricated SLM shows an approximate 30‐fold reduction in stiffness from rigid loading (pink) to compliant loading (purple). Measured data is shown as lighter lines with averages (n=5) shown as bold lines. e) The SLM is reprogrammed by pivoting in place and is parameterized by θ, the angle of the zero mode relative to the lattice connection direction. The SLM can be embedded onto 2D lattices using square symmetry (e) or two different types of hexagonal symmetry (f and g). Each of these configurations (e–g) is shown as CAD unit cell overlaid with its abstract lattice representation and FEA homogenized data shown in the polar plots below. Via in situ reprogramming, these materials can achieve substantially different extremal modes and resulting material properties without affecting the global lattice structure, such as transforming from a negative Poisson's ratio to a positive Poisson's ratio as shown in (e) or predicted (FEA) to support all four types of 2D isotropic extremal materials (f and g). Raw values for moduli are provided in Tables S3 and S4 (Supporting Information).

SLMs are the foundational element of our extremal materials. Among the various SLM designs,^[^ ^22^ ^]^ we devised a compliant double‐sided Robert's mechanism, inspired by prior work on generating a compliant single degree of freedom (DOF) motion^[^ ^26^ ^]^ (see **Figure** **1**a,b). They are compliant exclusively along their straight‐line DOF and rigid against orthogonal deformations, with an approximate 30‐fold decrease in stiffness between rigid and compliant loading shown in Figure 1c. SLMs are therefore inherently extremal, specifically unimodal, possessing one intrinsic zero mode. As shown by ref. [1] unimodes are sufficient to form the basis for all other extremal materials, thus our SLM‐based framework is well‐suited for extremal material construction.

A key innovation of our approach is the SLM's rotationally symmetric design, which allows in situ reorientation of the compliant direction (see Figure 1d); this tunes the zero modes of our extremal materials. The idealized SLM compliance can be expressed as the deformation gradient *F* of the SLM cell base and the subsequent linear strain $\varepsilon =\frac{1}{2}\left(F+F^{T}\right)-I$

(1) $$F=\left[\begin{array}{cc} 1+\alpha \cos\theta & 0\\ \alpha \sin\theta & 1 \end{array}\right]$$

and

(2) $$\varepsilon =\frac{\alpha }{2}\left[\begin{array}{cc} 2\cos\theta & \sin\theta \\ \sin\theta & 0 \end{array}\right]$$

where α is the non‐dimensional length scale of the SLM deformation and θ ∈ [0°, 180°) is the angle of the SLM DOF relative to the connective direction, shown in Figure 1d. The linear strain presented here is equivalent to the unimode strain expressed in Milton's and Cherkaev's foundational work.^[^ ^1^ ^]^ Thus, our SLM fulfills the original criterion for extremal material construction from unimode and can simultaneously adapt its extremal material response — from shear to normal deformation and back — through simple rotational adjustments. These unique characteristics enable in situ tuning of the mechanical behavior without requiring global structural change or lengthy reprogramming processes.

### 2.2 Zero Modes are Composable Using Planar Symmetry

Naïvely tiling unimodal SLMs can only result in unimodal materials with compliant strains described by Equation (2). Thus, introducing more DOF‐wise independent SLMs is necessary to achieve a broader range of behaviors; however, each adjoined SLM introduces constraints, (i.e., rigidity) along with its DOF as expressed by the zero value in Equation (2). Crucially, the DOFs and constraints must be coordinated to ensure that they do not interfere and restrict deformation, which requires simultaneously satisfying each individual SLM's deformation gradient. See Section S1 (Supporting Information) for more details.

We use 2D planar symmetries, called the *wallpaper groups*, to introduce multiple SLMs and coordinate their zero modes. Wallpaper groups ensure complete tiling of the plane for metamaterial construction.^[^ ^27^ ^]^ They also effectively arrange each individual SLM zero mode to avoid the combinatorial complexity seen in other efforts to design zero mode materials.^[^ ^10^ ^]^ This setup creates a 2D material where the number and characteristics of zero modes are determined by the SLM orientation and applied symmetry. Due to its uniform structure, our reprogrammable SLM fits seamlessly into regular square and hexagonal lattices. Significantly, in contrast to other reprogrammable zero mode materials,^[^ ^3^ ^]^ the overall global lattice structure is preserved regardless of the microstructure configuration (i.e., SLM orientation), demonstrated in Figure 1e–g. This allows the lattice to act as a scaffold, where the nodes serve as connection points for the SLMs positioned along the edges. The loading of the structure is transferred through the lattice connections, but deformations are allowed only in line with the SLM orientations. This results in a range of emergent behaviors based on a single lattice, which are coordinated by the orientation of zero modes relative to the lattice lines.

### 2.3 Analyzing Zero Modes

First, we developed an analytical model for the compliance matrix *S* = *C* ^−1^ using the idealized deformation gradient and linear strains (see Section  S2, Supporting Information for derivation). We then simulated the materials using finite element analysis (FEA) computational homogenization via ANSYS Material Designer (AMD) to extract an effective metamaterial linear elastic response across θ. Due to limitations of AMD, only orthogonal tilings are possible, precluding the ability to model the 632 material in the range of θ ∈ (0°, 90°). This allows us to compare theoretical models, fit data, and simulation for the full range of 2*22 and 442, but only the orthogonal modes of 632.

The square‐symmetry materials (2*22 and 442) were tested in different θ configurations using a 2 × 10 array under tensile and shear loading, with optical tracking to extract the engineering constants of the array (Young's moduli *E*_1_, *E*_2_, shear modulus *G*_12_, Poisson's ratios ν_*ji*_ = ‐ε_*i*_/ε_*j*_, and normal‐shear coupling η_12*k*_ = γ_12_/ε_*k*_). All mechanical tests were conducted with <2% global strain where linear elasticity is a reasonable approximation for extracting effective properties. The engineering constants were then used to recreate the full compliance matrix *S* = *C* ^−1^ for comparison with FEA results.

In order to directly compare between the FEA and measured results, we fit both sets of data to the analytical model using linear least‐square regression with non‐linear constraints (see Section S2, Supporting Information). This results in a more numerically robust and higher‐order model that characterizes the entire system across θ. In turn, we were able to investigate how the models behave holistically rather than on a point‐wise basis for each θ value, leading to more effective comparisons between FEA modeled and fabricated/simulated tests. We then analyzed the zero modes of these fitted models, unconvering relationships that would otherwise be obscured by numerical and measurement noise.

## 3 Results and Discussion

### 3.1 SLM Orientation and Symmetry Provide Different Emergent Properties

We observe a variety of emergent and extremal properties by constructing materials using wallpaper groups 2*22, 632, and 442 expressed here in the orbifold notation^[^ ^27^ ^]^ (cmm, p6, p4 in crystallographic notation) and shown in Figure 1e–g. In all cases, altering the orientation of the SLM within a single lattice type maintains the global structure, but leads to the development of distinct extremal materials.

Figure 1e shows the topological layout and FEA‐simulated data for three variations of the 2*22 symmetry for θ ∈ {0°, 45°, 90°}, which create three distinct orthotropic extremal materials: an auxetic bimode, a zero Poisson's ratio bimode, and a positive Poisson's ratio unimode. In each case, the behavior of the material is described by Equation (2) which prescribes the directions of compliance relative to the lattice lines.

The angles of Figure 1e show a wide range of simulated FEA properties. For θ = 0° the zero modes of a material will lie diagonally along the lattice lines, without any shear compliance. With SLMs along each diagonal of the lattice, this material is a bimode, compliant with extension along each of the diagonals independently. This is demonstrated in the moduli plot along the diagonal, where a low Young's modulus and a high shear modulus confirm the expected allowable deformations. Additionally, there is a zero Poisson's ratio along those diagonals with the DOFs, but the diverging nature of the DOFs causes a negative Poisson's ratio (auxetic) along the principal horizontal and vertical directions. The 2*22 θ = 45° structure is also a bimodal material, allowing independent horizontal and shear deformations due to the alignment of zero modes in the horizontal direction. This results in a low Young's modulus horizontally, a high Young's modulus vertically, a low shear modulus in the plane, and a zero Poisson's ratio in the plane. Finally, the 2*22 structure with θ = 90° exhibits unimodal properties. Complementary to the θ = 0° case, here only the off‐diagonal elements of Equation (2) are non‐zero, meaning that the material does not extend and can only shear relative to the lattice lines. This is seen with the low shear modulus along the diagonals. Furthermore, in contrast to θ = 0°, the DOFs are now aligned to produce a positive Poisson's ratio in the horizontal and vertical directions. Taken together, these three orientations prove that a single lattice can be dialed from stretch, to stretch‐shear, and then shear‐only behavior simply by rotating the SLMs, demonstrating full, on‐demand control over its deformation modes. Our analytical and FEA models indicate that hexagonal 632 symmetry can realize all four isotropic extremal types — null‐, uni‐, bi‐, and trimode — via two tiling variants (Figure 1f,g). Strikingly, the hexagonal lattices only need two orthogonal orientations (θ = 0° and 90°) to span this full isotropic extremal set. In this case the Type I tiling produces both nullmode and unimode materials (90° and 0° respectively), while Type II tiling produces bimode and trimode materials (90° and 0° respectively).

The Type I construction uses an equilateral triangle cell layout, which adds a geometric constraint that all sides must be equal upon deformation (see SI §1). The combination of this constraint and zero modes oriented perpendicular to the lattice lines (θ = 90°) results in a material that resists extension or shear deformation, making it a nullmode material. While nullmode materials are often considered trivial, our SLM‐based design produces a non‐trivial internal rotational motion due to the coordinated DOFs of its zero modes. Notably, this rotational DOF is fully described by the SLM deformation gradient (Equation 1), but is not captured in the linear elastic theory (Equation 2).

The Type I material with SLMs oriented along the lattice lines (θ = 0°) numerically demonstrates an isotropic unimode. The zero modes align with the lattice lines meaning that only expansion and contraction along the lattice dimensions are allowed. This produces a material that compresses and expands easily without shear, i.e., has a low bulk modulus and high shear modulus, a key feature of isotropic unimodes. Consequently, this also results in an FEA simulated negative Poisson's ratio of ν ≈ −0.88 which approaches the theoretical limit of −1 for isotropic 2D elastic materials.

Type II tiling covers the isotropic bimode‐trimode materials. Similar to the nullmode material, the bimode material has DOFs perpendicular to the lattice (θ = 90°); however, Type II tiling does not have the additional equilateral triangle constraint from Type I. Because of this, the bimode material is able to shear along the lattice lines, producing the opposite effect of the Type I unimode, that is a material with a high bulk modulus and low shear modulus. This bimode material is the 2D analog of the 3D pentamode material,^[^ ^1^, ^28^ ^]^ operating as a “metafluid,” resisting bulk compression, but compliant to shear or “flow.” Therefore, as expected, this material approaches the theoretical limit of +1 for isotropic 2D elastic materials with a simulated value of ν = 0.97.

Finally, the trimode material, like the nullmode, is normally considered trivial as it is completely compliant; however, our SLM trimode material has DOFs along the lattice lines (θ = 0°). As with the previous materials with DOFs along the lattice, this trimode material has a negative Poisson's ratio, a feature that has not been previously seen in other compliant extremal materials. The sum total of our isotropic materials is that we have shown a method of constructing all four types of isotropic extremal materials using SLMs.

### 3.2 The Property Space is Interpolative

In Figure 1 we see discrete states of the lattice; however, unlike previous work,^[^ ^3^, ^9^, ^28^ ^]^ our extremal materials are continuously reprogrammable. By rotating the SLMs we produce smooth variations in their properties. This produces an interpolative property space parameterized by the continuous variable θ ∈ [0, 180°). This variability arises from both the lattice symmetry and the SLM orientation, each independently influencing the material's properties. Thus, by using a single lattice we can enable or disable properties, like chirality or auxetics, through reprogramming of the SLM orientations.

**Figure 2:**
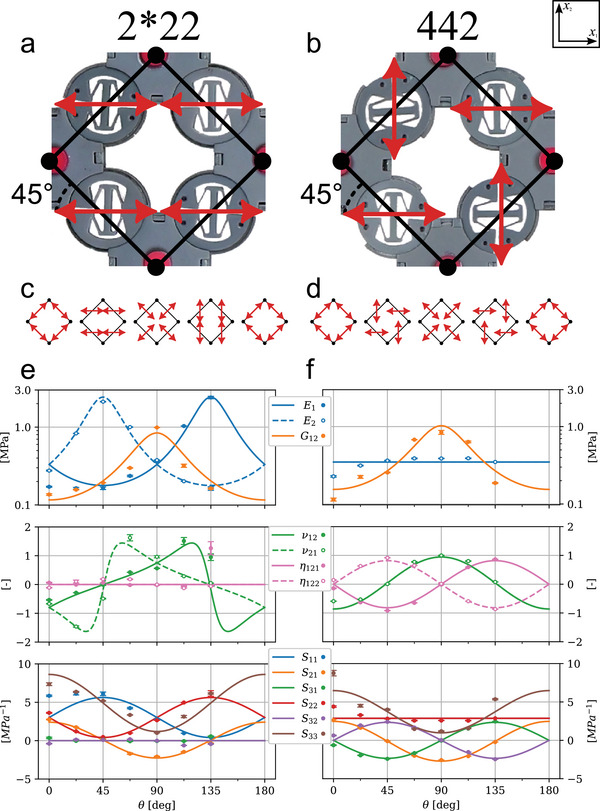
Fabricated 2*22 a) and 442 b) square symmetry materials shown in close‐up at a θ = 45° configuration. The illustrations below each image c,d) show the DOF alignment for each symmetry as a function of θ and correspond to the major x‐axis grid lines in the plots below. The data shown in e,f) show experimental data (dots) and corresponding analytical fits (solid/dashed lines). From top to bottom: the directional Young's and shear moduli (*E*_1_, *E*_2_ and *G*_12_); the Poisson's and normal‐shear coupling ratios (ν_12_, ν_21_ and η_121_, η_122_); and finally the individual *S* matrix components which were used to fit the model directly. Markers show the median of five repeats, n=5; vertical bars denote interquartile range. Data was not gathered for 157.5° due to limitations of the dovetail construction. Similarly, data was not gathered for 180° due to it having an identical construction to 0° and so was considered redundant.

The two materials shown in **Figure** **2** demonstrate how the same square lattice can produce distinct interpolative material property characteristics by enforcing different symmetry types (mirror vs. rotational) on the SLMs. Figure 2a,b shows the two square symmetry patterns, 2*22 and 442, in a θ = 45° configuration. Shown directly below (Figure 2c,d) is an illustration for how the DOFs differ due to mirror vs. rotational symmetry as θ varies is shown directly, which correspond to the x‐axis gridlines of the below plots. Finally, we show the measured data (dots) and fit model data (solid/dashed lines) in Figure 2e,f. The measured data was captured every 22.5° while the analytical model is fit to these points but interpolated along the full span of θ. Each θ configuration was tested with five physical replicates with median values reported and vertical bars representing the interquartile range (25th–75th percentiles). Data is not shown for 157.5° or 180°. Data was not captured for 157.5° due to limitations of the dovetail mechanism, while data for 180° was not captured due to being identical by construction to the 0° configuration and so was considered redundant in nature. Regardless, the physical performance is expected to follow the relationships driven by the symmetry of the material, predicted by the analytical model, and demonstrated numerically with the FEA data (see Figure S3, Supporting Information for FEA model fit).

For the 2*22 material in Figure 2e, the Young's modulus varies from low to high depending on the alignment of the SLMs, low when all the DOFs are aligned with a principal direction and high in the orthogonal direction, while the shear modulus peaks simply once for θ = 90°. Notably, where the Young's moduli peak (45° (*E*_2_) and 135° (*E*_1_)) the corresponding Poisson's ratio has an asymptotic behavior, due to the SLM rigid directions all being aligned in the respective principal direction, *x*_1_ or *x*_2_. While the shear modulus demonstrates a single peak at 90°, the normal‐shear coupling is minimal by the mirror symmetric nature of the 2*22 pattern, with the exception at 135°, which is an artifact of testing at the asymptotic point highlighted earlier for Poisson's ratio. Significantly, the moduli can be varied by an order of magnitude and along with that we see the Poisson's ratio vary from negative (auxetic) to positive.

In contrast to the 2*22 material, the 442 Young's modulus remains relatively flat (Figure 2f). This is expected because all four DOFs of the unit cell never fully align and, by rotating together, lead to a constant level of stiffness as a function of θ. The shear modulus, however, follows the same trend as the 2*22 material and also varies about one order of magnitude. Here the Poisson's ratio does not exhibit the asymptotic behavior, instead producing a cosine wave, while the normal‐shear couplings produce sine waves of opposite signs (η_121_ = −η_122_). This normal‐shear coupling is expected due to the rotational symmetry of the material which, lacking mirror symmetry, allows for non‐zero *S*_13_ and *S*_23_ values, a feature absent with the 2*22 material. Importantly, we see the chirality of the material changes as a function of θ with varying η_121_ from −1 to +1. This allows us to turn on and off the coupling as well as change the handedness of the material.

The bottom plot of Figure 2e,f shows the measured data and corresponding analytical model of the *S* matrix. The specific coefficients of the analytical model are then optimized to minimize the relative error across all *S* values equally and are provided in Table S2 (Supporting Information). Overall, there is good agreement between the analytical model and measured data with several key idealizations were made in generating the models. First for the 2*22 material, the values of *S*_31_ and *S*_32_ were assumed to be zero, due to the natural symmetry of the material. This is demonstrated in the analytically derived model and FEA data. While the data does not reflect zero values, it is still relatively small when compared to other values of *S*, most likely arising from noise in the motion capture system. For the 442 material, the values of *S*_11_ and *S*_22_ were assumed to be constant, again supported by the analytical model and corroborated by the FEA simulated data. These simplifying assumptions enable the model to capture the dominant trends in material behavior across all θ, while smoothing out local noise and inconsistencies in the measured data—resulting in a compact, robust representation that reflects both the symmetry‐driven physics of the system and the empirical observations.

### 3.3 The Extremal Materials Span the Eigenvalue Gamut

**Figure 3:**
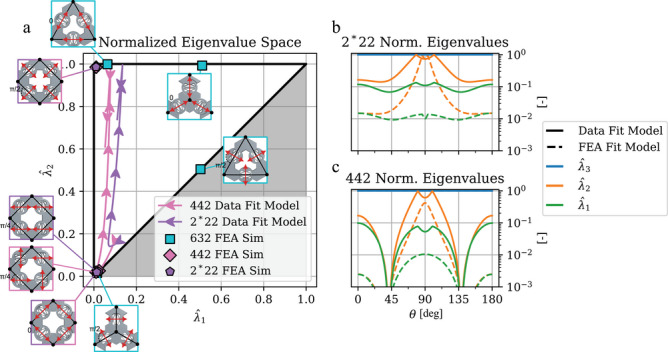
a) Normalized FEA simulated eigenvalues of the 2*22, 442 and 632 materials plotted as ${\hat{\lambda }}_{2}$ vs. ${\hat{\lambda }}_{1}$. Additionally, the transition from bimode to unimode of the fitted data models for the 2*22 (purple) and 442 (pink) are presented for θ = [0°, 90°]. The arrowheads indicate the direction of the trajectory as θ transitions from 0° to 90°. b,c) The normalized eigenvalues from the experimental data and FEA data analytical fits for the 2*22 and 442 materials respectively. The line style indicates the measured data and FEA models (solid and dashed) and the line color indicates the normalized eigenvalue being plotted for a given model (${\hat{\lambda }}_{3}$ is blue, ${\hat{\lambda }}_{2}$ is orange, ${\hat{\lambda }}_{1}$ is green). Absolute and relative eigenvalues for all FEA points are tabulated in Table S4 (Supporting Information).

To analyze the transitions of SLM lattices, we define the “extremal material gamut” (**Figure** **3**) which provides a stiffness‐ and coordinate‐invariant space to compare materials. Here we use normalized eigenvalues of *C* sorted smallest to largest, constraining the values within the upper‐left triangle of the unit square. Specifically, if the eigenvalues of the 3 × 3 *C* are 0 < λ_1_ ⩽ λ_2_ ⩽ λ_3_ then the normalized eigenvalues are ${\hat{\lambda }}_{i}=\lambda_{i}/\lambda_{3}$ for *i* = 1, 2, 3. Because ${\hat{\lambda }}_{3}=1$ by definition, we plot the relationships in a 2D space ${\hat{\lambda }}_{2}$ vs. ${\hat{\lambda }}_{1}$. In this space, the diagonal represents λ_2_ = λ_1_ while the top line represents λ_2_ = λ_3_. The remaining left‐most boundary forms the extremal boundary, where there is always one small relative eigenvalue (λ_1_ ≪ λ_3_) and defines the ideal unimode and bimode materials at points (0, 1) and (0, 0) respectively. Importantly, because we normalize each material configuration at θ to *its own* largest eigenvalue we can compare unimode and bimode materials directly in terms of their extremal characteristics independent of their inherent stiffness. The tradeoff for this normalization is that it obscures materials with three small, but relatively close, eigenvalues, such as the 632 trimode discussed below.

As shown in Figure 2, just as the material properties smoothly vary over θ, so do the eigenvalues. This is seen by the purple and pink trajectories for the measured‐data model in Figure 3a. The significance of the trajectories is two‐fold: 1) both material sets (2*22 and 442) maintain relative extremality and 2) the extremal space is continuously variable. Here, the trajectories span θ = 0° to θ = 90° and follow the flow of the arrows. Figure 3b and c show the normalized eigenvalues of these trajectories as a function of θ. In each case the analytical 2*22 and 442 materials consistently have one small eigenvalue $\left({\hat{\lambda }}_{1}≲10^{-1}\right)$ while ${\hat{\lambda }}_{2}$ varies from low to high, nearly equal with ${\hat{\lambda }}_{3}$ as θ → 90°. This means the trajectories in the gamut (Figure 3a) show the square‐symmetry materials transitioning from bimode to unimode by tracking along the far‐left extremal boundary. The back‐tracking seen toward the end of these trajectories, while remaining extremal, indicates a reordering of the eigenvalues relative to the eigenvectors, which is an artifact of the eigenvalue normalization process.

Beyond the measured‐data model, we fit the FEA simulated data in the same manner for direct comparison. These are represented as the scatter data points on Figure 3a and the dashed lines in Figure 3b,c. Here we see all the examples from Figure 1e–g, now shown in an extremal landscape. The 2*22 and 442 θ = 0° and θ = 45° materials are all bimodal, existing at the bottom left corner, noting that the 2*22 and 442 θ = 0° materials are identical in DOFs and so overlap here. The 2*22 and 442 θ = 90° material (again identical in DOFs) is shown at the top‐left corner clearly exhibiting unimodal characteristics. Significantly, this demonstrates that the square‐symmetric materials are capable of maintaining extremality while transitioning from bimode to unimode.

The 632 materials from Figure 1f and g are all shown. The null mode material (Type I tiling, θ = 0°) lies plainly in the middle of the space with no small eigenvalues approximately at (0.5, 0.5). For the extremal materials, the isotropic unimode (Type I tiling, θ = 0° lies at the to left corner, while the bimode (Type II tiling, θ = 90°) lies at the bottom left corner, matching their desired extremal behavior. Notably, the trimode material (Type II tiling, θ = 0°) does not lie on an extremal boundary. While counterintuitive, this is an artifact of the normalization process. This gamut obscures materials where all the eigenvalues are small relative to the base material, as in the 632 trimode case, which lies near (0.5, 1) despite have three simulated small eigenvalues, as shown Table S3 (Supporting Information). Regardless, the fact that all the unimode and bimode materials lie in their respective domains, and that the trajectories of the square‐symmetric materials track along the extremal boundary, confirms the desired outcome of 1) programming all types of extremal behavior and 2) the ability to smoothly interpolate between extremal points.

## 4 Spatially Varying Orientations Make Programmable Composites

**Figure 4:**
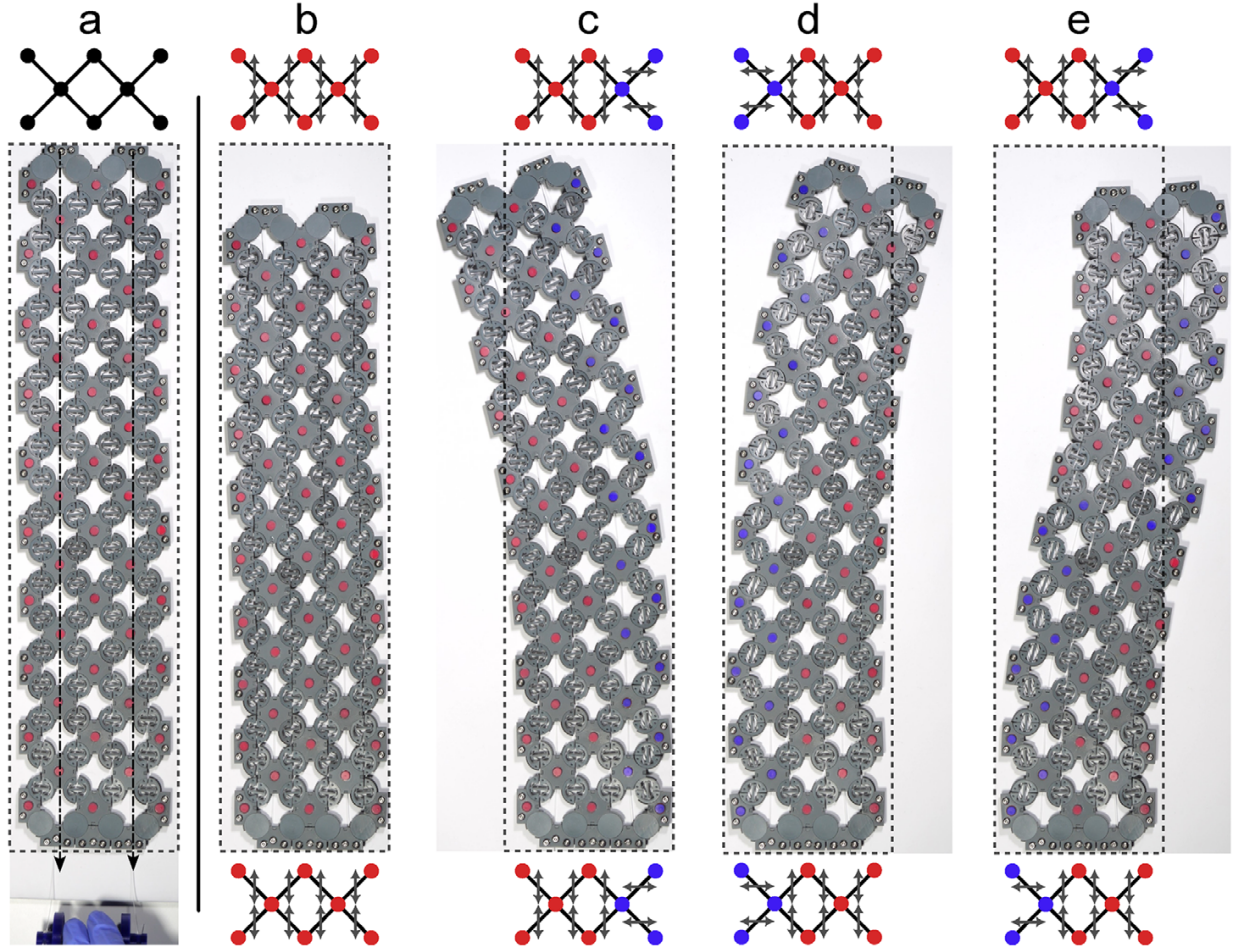
A single, cable‐driven array can be spatially reprogrammed to provide unique actuation modes. a) depicts an illustration of the lattice structure in the undeformed state while b–e) show deformed states. Markers are physically red; in each panel we recolor subsets to match the DOF groupings shown in the lattice illustrations. SLM orientations are depicted by gray arrows. (b) has all the SLMs oriented vertically and compresses with ν ≈ 0 under tension. (c) and (d) have one column of horizontally oriented SLMs, right and left respectively causing the lateral bends. (e) uses different SLM orientations on the top and bottom halves of the array to create the composite S‐bend.

To demonstrate the utility of the reprogrammable SLM framework we implemented a 2D column with spatially tailored material properties. By globally coordinating the changes in the angle θ of the SLMs, we control the properties of the entire lattice; however, the angle θ also provides us with a field to spatially vary the local properties of the lattice. We take advantage of this tunability by reprogramming local stiffness in a finite array to provide different modes of actuation, as shown in **Figure** **4**.

We utilized our 2 × 10 array from measurement characterization to spatially compose different stiffnesses by varying SLM alignment throughout the structure. To load the array, we affixed two cables at the top, routed them downward through the body, and connected them to the array throughout the length to prevent buckling (Figure 4a).

When all SLMs are aligned with the loading direction (Figure 4b), the material compresses in the vertical direction. This lattice has the properties of the 2*22 material previously described in Figure 2a for θ = 135°; namely low *E*_2_ and ν_21_ ≈ 0).

In contrast, reprogramming one column of SLMs perpendicularly partially emulates the 2*22 material for θ = 45° along that vertical band (i.e., high *E*_2_). The outcome of the striping process is a material that exhibits low stiffness on one side and high stiffness on the other in relation to the loading direction. Notably, because both of these configurations have low and equal shear modulus (seen in Figure 2e) the material is allowed to bend easily without significant shear stresses. This results in lateral bends as only one side of the array compresses, and by selecting the right or left most column for stiffening, we can control the direction in which the array bends (Figure 4c,d).

Leveraging the spatial reprogrammability even further, the design allows for more sophisticated behaviors, as illustrated by the S‐bend curve pattern in Figure 4e. We take the lattice in Figure 4a, split it vertically into two halves, and program the upper half like Figure 4c and the bottom like Figure 4d. This leads to an S‐shaped curve when compressed with complementary bending. The adaptability shows that our SLM‐based materials enable the development of spatially programmable mechanical metamaterials within a single lattice and furthermore is not restricted to simple configurations. Significantly, a myriad of behaviors including auxetics and chirality are technically achievable as long as the DOFs and constraints are resolved without frustration.

## 5 Conclusion 

In this study, we introduced a novel, reprogrammable approach to engineering 2D extremal materials using SLMs and planar symmetry. SLMs provide a geometric basis for explicitly programming the zero modes of extremal materials, enabling experimental construction for square lattices and numerical validation for hexagonal lattices across nullmode through trimode responses, dictated by SLM orientation (θ), lattice type (square vs. hexagonal), and orientation symmetry (rotational vs. mirror). By controlling the SLM orientation, we can spatially vary elastic properties across a given lattice, allowing selective tuning of Poisson's ratio, bulk‐to‐shear stiffness ratio, and chirality within a unified framework. We demonstrated the breadth of this approach by simulating and experimentally validating the square‐symmetric unimode and bimode emergent material properties and extremal behaviors a stiffness‐ and coordinate‐invariant extremal material gamut. We also demonstrate numerically how two types of hexagonal tilings can produce all four types of 2D isotropic extremal materials. This work not only realizes distinct extremal states but also demonstrates smooth, continuous transitions between them, representing the first such achievement in extremal materials.

While our results demonstrate the ability of a single reconfigurable unit to access all four extremal deformation classes, practical limitations remain. Our simulations were primarily restricted to rectangular tilings due to ANSYS limitations with non‐rectilinear periodic boundary conditions, leading us to fabricate only square‐symmetric specimens. Future work will experimentally confirm the predicted continuous behavior of hexagonal specimens. Although experiments were conducted within the linear elastic regime (under 2% global strain), local large strains, geometric nonlinearities, buckling, or friction were not explicitly modeled and may influence behavior at larger deformations. This reliance on standard linear elasticity also meant we could not fully validate intriguing DOFs, such as the internal rotational motion observed rotationally symmetric materials. These uncaptured behaviors suggest a promising direction for future theoretical and experimental work exploring connections to micropolar/Cosserat elasticity and their potential for phenomena like mechanical cloaking.^[^ ^5^, ^6^ ^]^ Finally, long‐term durability and fatigue resistance of compliant SLM joints were not characterized. Future efforts will address material fatigue, creep, and statistical variation across printed samples through higher‐throughput testing and extended mechanical cycling to validate performance in real‐world scenarios.

This work significantly expands the design space for mechanical metamaterials, enabling geometrically intuitive, on‐demand, dynamic adjustments of mechanical properties without structural alterations or costly computational methods. The continuous reprogrammability and arbitrary construction of zero modes unlocked by our SLM‐based framework offer unprecedented control over material behavior. This paves the way for practical applications demanding adaptive mechanical responses, from next‐generation soft robotics and underwater acoustics to advanced wearable technologies, fundamentally shifting how we design and utilize materials with tailored mechanical properties.

## 6 Experimental Section

### Fabrication

All SLM samples were fabricated using a Carbon 3D M1 printer with UMA‐90 material using standard print settings. The SLMs were printed in plane with the print bed and with a Carbon 3D film release coating applied to the print bed. Post‐processing followed the manufacturer's recommendations.

### Simulation

Computational homogenization was performed using ANSYS Material Designer. Representative volume elements (RVEs) were created in CAD software and simulated with periodic boundary conditions in 3D to extract the full 6 × 6 3D elasticity matrix. This matrix was then reduced to the 3 × 3 elasticity matrix for 2D plane stress materials. In this work, all analysis of simulated data was done on the reduced 2D matrix.

RVEs for 2*22 and 442 materials were generated to simulate data for θ ∈ [0°, 180°) in 5° steps, using symmetry to minimize the required number of simulations. 632 models were simulated with only θ = 0° and 90° due to ANSYS Material Designer's limitations of 1) rectilinear periodic boundary conditions and 2) the entire simulated body needing to be one continuous object.

### Test and Characterization

A 2 × 10 square‐lattice array was fabricated to validate and characterize the 2*22 and 442 materials. The design incorporates snap‐fit connectors and a rotational dovetail, facilitating reconfigurable and easy assembly. This modular construction allows for the easy replacement of parts because the SLMs are not rigidly connected, instead relying on friction and other forces to maintain contact. A ratcheting scheme aligns and secures the SLMs during testing. See Section S4 and Figures S3 and S4 (Supporting Information) for more details.

The array was tested on a universal testing machine (UTM) (Instron 5965) in a quasi‐static manner. For each rotation angle five tests were performed; the median value, with error bars spanning the interquartile range (25th–75th percentiles), were reported. Two symmetry patterns (2*22 and 442) were tested, in two different loading conditions (tensile and shear), with approximately five tests per θ value for θ ∈ 0, 22.5°, 45°, 67.6°, 90°, 112.5°, 135°. Measured data could not be captured for θ = 157.5° due to the dovetail mechanisms construction and data at θ = 180° is identical to 0° by construction, thus this data is not included in the measured data.

The force vs. displacement curves and videos of the array deforming array were recorded for testing at various θ configurations. The global force was used for modulus characterization (*E*_1_, *E*_2_, *G*_12_), while the middle third of the optical trackers were used to capture deformation behavior and characterize strain coupling ratios (ν_12_, ν_21_, η_121_, η_122_). The middle third of the trackers were used to avoid clamped boundary effects at the top and bottom end of the array.

## Conflict of Interest

The authors declare no conflict of interests.

## Supporting information

Supporting Information

## Data Availability

The data that support the findings of this study are openly available at https://bit.ly/SLM‐Data.
