# Supplementary material for: Design and Reprogrammability of Zero Modes in 2D Materials from a Single Element
Source: Adv Sci (Weinh). 2025 Aug 20;12(38):e11227. doi: 10.1002/advs.202511227 (PMC12520473; doi:10.1002/advs.202511227)
Supplement: Supplementary file 1 — Supporting Information [file ADVS-12-e11227-s001.pdf]

ADVANCED SCIENCE
Open Access

Supporting Information

for *Adv. Sci.*, DOI 10.1002/advs.202511227

Design and Reprogrammability of Zero Modes in 2D Materials from a Single Element

*Daniel Revier, Molly Carton and Jeffrey I. Lipton**

Design and Reprogrammability of Zero Modes in 2D Materials from a Single Element: Supporting Information

*Daniel Revier Molly Carton Jeffrey I. Lipton**

Daniel Revier
Paul G. Allen School of Computer Science and Engineering, University of Washington, Seattle, WA, 98195
Email Address: drevier@uw.edu

Molly Carton
Mechanical Engineering, University of Maryland, College Park, MD, 20742
Email Address: mcarton@umd.edu

Jeffrey I. Lipton
Mechanical and Industrial Engineering, Northeastern University, Boston, MA, 02115
Email Address: j.lipton@northeastern.edu

1 Straight Line Mechanism Deformation Gradients and Strains

Single SLMs

We analyze the behavior of the SLM extremal materials in two ways: through the deformation gradient and through the linear strain. We first express the idealized SLM compliance as the deformation gradient $\mathbf{F}$ of the SLM cell base as seen in the main text Figure 1a

$$\mathbf{F} = \begin{bmatrix} 1 + a\cos\theta & 0 \\ a\sin\theta & 1 \end{bmatrix}, \tag{S1}$$

where a is the length scale of the SLM deformation and $\theta \in [0, \pi)$ is the angle of the SLM DOF (red arrow) relative to the connective direction (black solid line). We can then express the SLM's zero mode as a linear strain

$$\varepsilon = \frac{1}{2}(\mathbf{F} + \mathbf{F}^T) - \mathbf{I} = \frac{a}{2}\begin{bmatrix} 2\cos\theta & \sin\theta \\ \sin\theta & 0 \end{bmatrix} \tag{S2}$$

which is equivalent to the unimode material described in [1] Equation 2.6.
Notably, the SLM and its deformation gradient exhibit rotational symmetry (C2 point group), whereas the strain shows mirror symmetry (D2 point group) for θ values of 0 and 90 degrees. Therefore, even if the symmetry pattern is solely rotationally symmetric, such as 632, the material properties can display characteristics requiring mirror symmetry, like isotropy.

Lattice and Deformation Vector Construction

A general method of constructing compliant extremal materials and analytically determining their zero modes is developed and illustrated in Figure S1 and algebraically shown in the supplementary file `extremal_alg`
We assume periodic tiling using the lattice vectors $\mathbf{L} = \begin{bmatrix} \hat{l}_1 & \hat{l}_1 \end{bmatrix}$. We are free to choose the orientation of our coordinate system and make $\hat{l}_1 = [1, 0]^T$ for simplicity. All other vectors (lattice and deformation) can be derived as various rotations of this vector. We find the Cartesian deformation gradient $\mathbf{F}$ for each unit cell and then can analyze unique deformation modes as well as the linear strain zero modes.
The generalized lattice vectors are defined with $\hat{l}_1$ oriented along the x-axis. The second lattice vector $\hat{l}_2$ is generated by rotating $\hat{l}_1$ by ϕ radians

$$\hat{l}_2 = \mathbf{R}(\phi)\hat{l}_2 = \begin{bmatrix} \cos\phi \\ \sin\phi \end{bmatrix}$$

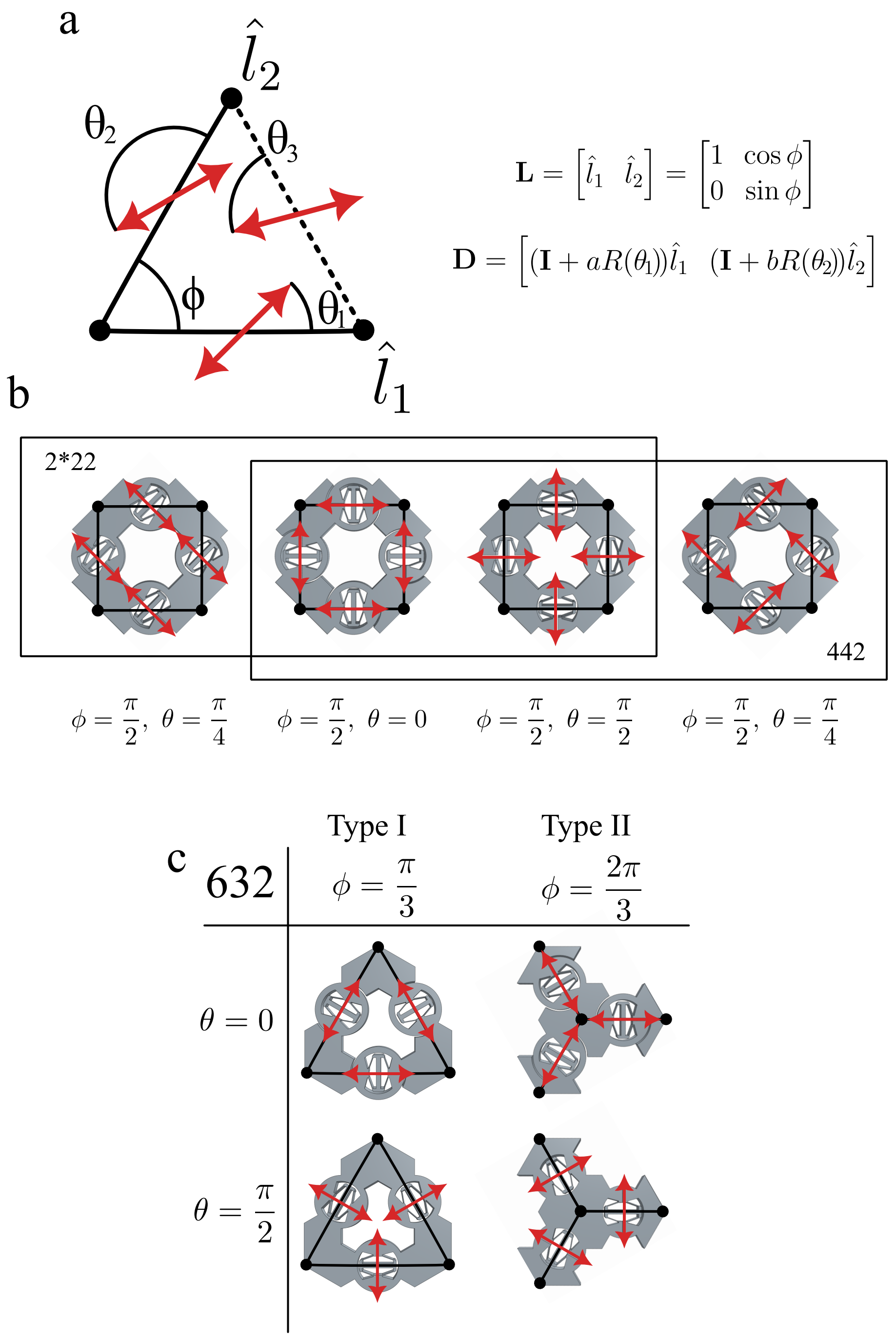

Figure S1: The generalized form of constructing SLM lattices. a) The two lattice vectors form matrix $\mathbf{L}$ and the deformation gradient, parameterized by a and b is found as rotational offsets from the lattice vectors. Under symmetry the SLM rotation angle θ is assumed to be the same for every SLM. b) The two square symmetries use $\phi = \frac{\pi}{2}$ for their lattice construction, but vary in how the symmetry is applied to realize the other SLM orientations. They are identical for $\theta = 0, \frac{\pi}{2}$ where they converged into *442 symmetry. c) 632 symmetry uses two different lattice vector sets, $\phi = \frac{\pi}{3}, \frac{2\pi}{3}$, for Type I and Type II cells respectively. Unlike the square symmetry case there is an additional SLM to account for in the deformation gradient.

Table S1: Symmetry enforces different constraints on the lattice shown here for the four types of symmetry explored in this work. θ_1 is the driven design parameter that defines all other SLM orientations. Square symmetry was restricted to tiling only two SLMs, making θ_3 undefined for 2*22 and 442 symmetry.

	2*22	442	632 - Type I	632 - Type II
ϕ	$\frac{\pi}{2}$	$\frac{\pi}{2}$	$\frac{\pi}{3}$	$\frac{2\pi}{3}$
θ_2	-θ_1	θ_1	θ_1	θ_1
θ_3	-	-	θ_1	θ_1

where $\mathbf{R}(\phi)$ is the standard 2D rotation matrix

$$\mathbf{R}(\phi) = \begin{bmatrix} \cos\phi & -\sin\phi \\ \sin\phi & \cos\phi \end{bmatrix}$$

(Figure S1a). Square lattices used in this work have $\phi = \frac{\pi}{2}$ and hexagonal lattices have $\phi = \frac{n\pi}{3}$ where $n = 1, 2$ (Figure S1c). For 632 symmetry, a third vector $\hat{l}_3$ must be considered to account for additional DOFs and constraints, which can be incorporated as terms into the first two.
The deformation vector of an SLM is a rotation of the lattice vector it is defined on

$$d_i = \mathbf{I} + \xi\mathbf{R}(\theta_i)\hat{l}_i$$

where ξ represents the free parameters associated with that SLM. The deformation gradient of an entire unit cell is then

$$\mathbf{D} = \begin{bmatrix} d_1 & d_2 \end{bmatrix} = \begin{bmatrix} (\mathbf{I} + a\mathbf{R}(\theta_1)\hat{l}_1) & (\mathbf{I} + b\mathbf{R}(\theta_2)\hat{l}_2) \end{bmatrix}. \tag{S3}$$

The SLM deformation vectors are defined by the individual θ_i for $i = 1, 2, 3$. For $\theta_i = 0$ the SLM DOF is aligned with the lattice vector it is attached to. The allows us to again use a rotation of the lattice vector to define the deformation vectors d_1 and d_2.
The deformation vectors have the free parameters a and b for square symmetry as well as c in 632 symmetry. These parameters are assumed to lie within the interval [0, 1] and represent the length scale of displacement of the DOF. The unit cell deformation gradient must simultaneously satisfy all individual SLM DOFS and constraints.
The deformation gradient $\mathbf{D}$ expresses global deformations in terms lattice vector lines, which may not necessarily be the global Cartesian space. In general, global deformation gradient can be found from the lattice deformation gradient

$$\mathbf{F} = \mathbf{L}\mathbf{F}'\mathbf{L}^{-1}$$

where $\mathbf{F}'$ is the deformation gradient of the lattice in lattice coordinates and the lattice coordinates $\mathbf{L}$ work as transformation matrices between the two. $\mathbf{D}$ already expresses global deformation in lattice coordinates $\mathbf{D} = \mathbf{L}\mathbf{F}'$, so to find the Cartesian deformation gradient we use

$$\mathbf{F} = \mathbf{D}\mathbf{L}^{-1}. \tag{S4}$$

This approach allows us to articulate deformations in a universal coordinate system, thereby generalizing the model to accommodate various lattice symmetries and configurations.

Applying Symmetry

Applying symmetry introduces constraints to the generalized lattice and deformation construction seen in Table S1. Pure rotational symmetry uses the lattice construction with the constraint $\theta_i = \theta_j$, $\forall i, j$. Mirror symmetry has the constraint of $\theta_2 = -\theta_1$, but for mirror symmetry there is no way to define θ_3 and be mirror symmetric with both θ_1 and θ_2. This means that the 632 wallpaper group can not, in general, produce mirror symmetric deformation gradients across θ, which is observed on the extremal gamut. The trajectory of an isotropic extremal material from one state to another passes through the anisotropic regime, implying that isotropy is not allowed while maintaining symmetry.

Analyzing the Deformation Gradient

We can construct an idealized deformation gradient for the unit cells by linearly combining multiple SLM deformation gradients (Equation S1) and resolving any constraints. This is shown in Figure S2 and derived in `extremal_algebra.m`, where the deformation gradient is derived from the generalized lattice formulation in each case. There are two independent SLMs in square symmetry (red and blue) and three independent SLMs for hexagonal symmetry (red, blue, and green). These are color coded to highlight how each SLM is allowed to independently deform and illuminate the relationship with the deformation gradient.

For example, Figure S2a is the 2*22 $\theta = 0$ bimodal material. This material allows horizontal deformation along the horizontal lattice lines and vertical deformation along vertical lattice lines. This is shown as the independent red and blue arrows, but also conveyed in the deformation gradient with the independent parameters a and b each allowed to modify the deformation gradient. The Type I hexagonal materials have a third SLM which operates as a constraint on the unit cell by ensuring $|\hat{l}_1| = |\hat{l}_2|$. Thus, the deformation gradient actually loses free parameters, seen in Figure S2e and f having only the free parameter $a = b$. Meaning, that if the SLM that corresponds to a deforms then then SLM corresponding to b must also deform.

Analyzing the deformation gradient directly can provide insight into the material that is lost in linear elasticity. For example, the isotropic nullmode material (Figure S2e) has deformation gradient

$$\begin{bmatrix} 1 & -a \\ a & 1 \end{bmatrix}$$

indicating rotation that is possible in the unit cell. The eigenvectors (Q) and eigenvalues (Λ) of this material's deformation gradient are

$$Q = \begin{bmatrix} -i & i \\ 1 & 1 \end{bmatrix}, \quad \Lambda = \begin{bmatrix} 1 - ai & 0 \\ 0 & 1 + ai \end{bmatrix}$$

confirming that rotational deformation of the material is possible. However, this information is lost in the translation to Cauchy elasticity which does not account for rotations. This is seen by the compliant strain equaling the zero vector in Figure S2, seemingly indicating that no deformation is allowed. The use of polar DOFs is of substantial interest [2, 3, 4] and an examination using micropolar or Cosserat elasticity theories would be required to explore this concept further.

The compliant linear strain provides key insight into a material's behavior by revealing the zero modes of a linear elastic material. We find the zero modes by converting the deformation gradient to strain which, in the limit of infinite compliance, satisfies

$$\sigma = 0 = C\varepsilon \tag{S5}$$

implying that the compliant strains form the nullspace of C. In reality, the elasticity matrix C is always symmetric positive definite, meaning that Equation S5 can only be satisfied when $\varepsilon = [0, 0, 0]^T$. However, if the compliance is weak enough it will still approximately satisfy Equation S5. Equivalently, the compliant strains are the eigenvectors corresponding to zero-value eigenvalues, meaning that the number of independent zero-energy strain vectors (i.e., the nullity of C) are the extremal mode of the material. This is seen in Figure S2, where nullmode materials have no eigenstrains (outside the trivial zero vector), unimodal materials have one eigenstrain, bimodal materials have two, and the trimodal materials have three.

2 An Analytical Model for the Compliance Matrix S

Derivation

The analytical compliance matrix can be developed using the same linear strain defined in Equation S2. We express the Voigt strain $\varepsilon = [\varepsilon_{xx}, \varepsilon_{yy}, \gamma_{xy}]^T$ in terms of the strain-DOF operator matrix $B(\theta)$ and the

a b c		Deformation Gradient (**F**)	Compliant Strains (ε)	Eigenstrains
a	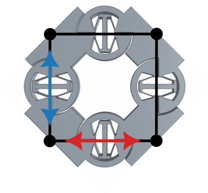	$\begin{bmatrix} 1+a & 0 \\ 0 & 1+b \end{bmatrix}$	$\begin{bmatrix} a \\ b \\ 0 \end{bmatrix}$	$\begin{bmatrix} 1 \\ 0 \\ 0 \end{bmatrix}, \begin{bmatrix} 0 \\ 1 \\ 0 \end{bmatrix}$
b	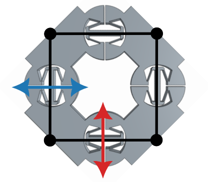	$\begin{bmatrix} 1 & a \\ b & 1 \end{bmatrix}$	$\begin{bmatrix} 0 \\ 0 \\ a-b \end{bmatrix}$	$\begin{bmatrix} 0 \\ 0 \\ 1 \end{bmatrix}$
c	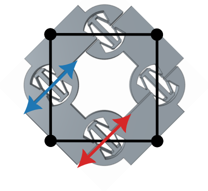	$\begin{bmatrix} a+\frac{a}{\sqrt{2}} & \frac{b}{\sqrt{2}} \\ \frac{a}{\sqrt{2}} & 1+\frac{b}{\sqrt{2}} \end{bmatrix}$	$\begin{bmatrix} \frac{\sqrt{2}a}{2} \\ \frac{\sqrt{2}b}{2} \\ \frac{\sqrt{2}a}{2}+\frac{\sqrt{2}b}{2} \end{bmatrix}$	$\begin{bmatrix} \frac{1}{\sqrt{2}} \\ 0 \\ \frac{1}{\sqrt{2}} \end{bmatrix}, \begin{bmatrix} 0 \\ \frac{1}{\sqrt{2}} \\ \frac{1}{\sqrt{2}} \end{bmatrix}$
d	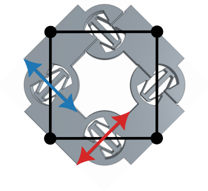	$\begin{bmatrix} a+\frac{a}{\sqrt{2}} & \frac{-b}{\sqrt{2}} \\ \frac{a}{\sqrt{2}} & 1+\frac{b}{\sqrt{2}} \end{bmatrix}$	$\begin{bmatrix} \frac{\sqrt{2}a}{2} \\ \frac{\sqrt{2}b}{2} \\ \frac{\sqrt{2}a}{2}-\frac{\sqrt{2}b}{2} \end{bmatrix}$	$\begin{bmatrix} \frac{1}{\sqrt{2}} \\ 0 \\ \frac{1}{\sqrt{2}} \end{bmatrix}, \begin{bmatrix} 0 \\ \frac{1}{\sqrt{2}} \\ -\frac{1}{\sqrt{2}} \end{bmatrix}$
e	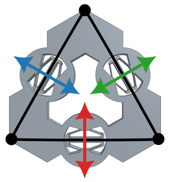	$\begin{bmatrix} 1 & -a \\ a & 1 \end{bmatrix}$	$\begin{bmatrix} 0 \\ 0 \\ 0 \end{bmatrix}$	$\begin{bmatrix} 0 \\ 0 \\ 0 \end{bmatrix}$
f	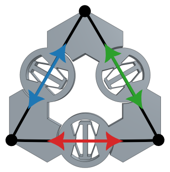	$\begin{bmatrix} 1+a & 0 \\ 0 & 1+a \end{bmatrix}$	$\begin{bmatrix} a \\ a \\ 0 \end{bmatrix}$	$\begin{bmatrix} \frac{1}{\sqrt{2}} \\ \frac{1}{\sqrt{2}} \\ 0 \end{bmatrix}$
g	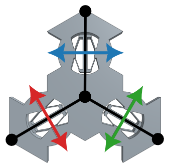	$\begin{bmatrix} 1+\frac{\sqrt{3}c}{6}-\frac{\sqrt{3}a}{6} & -\frac{a}{6}-\frac{2b}{3}-\frac{c}{6} \\ \frac{a}{2}+\frac{c}{2} & 1+\frac{\sqrt{3}a}{6}-\frac{\sqrt{3}c}{6} \end{bmatrix}$	$\begin{bmatrix} -\frac{\sqrt{3}(a-c)}{6} \\ \frac{\sqrt{3}(a-c)}{6} \\ \frac{a}{3}-\frac{2b}{3}+\frac{c}{3} \end{bmatrix}$	$\begin{bmatrix} -\frac{1}{\sqrt{2}} \\ \frac{1}{\sqrt{2}} \\ 0 \end{bmatrix}, \begin{bmatrix} 0 \\ 0 \\ 1 \end{bmatrix}$
h	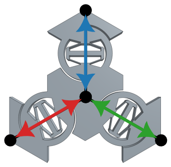	$\begin{bmatrix} 1+\frac{a}{2}+\frac{c}{2} & \frac{\sqrt{3}(a-c)}{6} \\ \frac{\sqrt{3}(a-c)}{6} & 1+\frac{a}{6}+\frac{2b}{3}+\frac{c}{6} \end{bmatrix}$	$\begin{bmatrix} \frac{a}{2}+\frac{c}{2} \\ \frac{a}{6}+\frac{2b}{3}+\frac{c}{6} \\ \frac{\sqrt{3}(a-c)}{3} \end{bmatrix}$	$\begin{bmatrix} 1 \\ 0 \\ 0 \end{bmatrix}, \begin{bmatrix} 0 \\ 1 \\ 0 \end{bmatrix}, \begin{bmatrix} 0 \\ 0 \\ 1 \end{bmatrix}$

Figure S2: The three symmetry groups examined in this study are presented at specific angles in Table S1. Deformation vectors are indicated by colored arrows, with a legend above explaining the correspondence of each vector to the variables a, b, or c. Deformation gradients are calculated by fulfilling all independent SLMs within the cell concurrently. Compliant strains are determined as linear strains and are displayed in vector form. These strains can also be expressed as linearly independent eigenstrains, which are the unique strains compliant with a material constructed with the specified DOF.

DOFs $q = [a, b]^T$ where a and b are the DOFs for the individual SLMs shown in Equation S3 as

$$\varepsilon(\theta) = B(\theta)q. \tag{S6}$$

$B(\theta)$ then dictates the allowable deformation modes of the lattice irrespective of the actual SLM deformation length scales. We then model each SLM stiffness with an axial spring k_i, letting $K = \text{diag}(k_a, k_b)$. Minimizing the internal energy $U = \frac{1}{2}q^T K q$ subject to the imposed strain leads to $q = K^{-1}B^T\sigma$ and therefore

$$S(\theta) = B(\theta)K^{-1}B(\theta)^T \tag{S7}$$

which is the effective compliance matrix seen at the macroscale. If all the SLMs are equal stiffness ($k_i = k$) Equation S7 reduces to $S(\theta) = \frac{1}{k}B(\theta)B(\theta)^T$.

Fitting Analytical Models to Data

Analytical models were fit to both the measured data and the FEA square-symmetric (2*22 and 442) simulated data for direct comparison. This provides a robust and comprehensive method of directly comparing the material characteristics and behavior across θ rather than on a point-by-point basis. First, the analytical models were derived for the 2*22 and 442 materials in the lattice orientation used for testing and simulation. Assuming $k_i = k = 1$, the characteristic behaviors are then

$$S(\theta)_{2*22} = \begin{bmatrix} \frac{1}{2}\left(1+\sin(2\theta)\right) & \frac{1}{2}\cos(2\theta) & 0 \\ \frac{1}{2}\cos(2\theta) & \frac{1}{2}(1-\sin(2\theta)) & 0 \\ 0 & 0 & \cos(2\theta)+1 \end{bmatrix} \tag{S8}$$

and

$$S(\theta)_{442} = \begin{bmatrix} \frac{1}{2} & \frac{1}{2}\cos(2\theta) & -\frac{1}{2}\sin(2\theta) \\ \frac{1}{2}\cos(2\theta) & \frac{1}{2} & \frac{1}{2}\sin(2\theta) \\ -\frac{1}{2}\sin(2\theta) & \frac{1}{2}\sin(2\theta) & \cos(2\theta)+1 \end{bmatrix}. \tag{S9}$$

We generalize the models to optimize over a set of linear coefficients C

$$S(\theta)_{2*22}^{opt} = \begin{bmatrix} c_1\sin(2\theta)+c_2 & c_3\cos(2\theta)+c_4 & 0 \\ c_3\cos(2\theta)+c_4 & c_2-c_1\sin(2\theta) & 0 \\ 0 & 0 & c_5\cos(2\theta)+c_6 \end{bmatrix} \tag{S10}$$

and

$$S(\theta)_{442}^{opt} = \begin{bmatrix} c_1 & c_2\cos(2\theta)+c_3 & c_4\sin(2\theta) \\ c_2\cos(2\theta)+c_3 & c_1 & -c_4\sin(2\theta) \\ c_4\sin(2\theta) & -c_4\sin(2\theta) & c_5\cos(2\theta)+c_6 \end{bmatrix} \tag{S11}$$

optimizing over all $c_i \in C$ for each material set independently. The optimization function then becomes

$$\begin{aligned} \min_{C} \quad & \sum_{\theta_i\in\Theta} \frac{(S(\theta_i;C)^{\text{opt}} - \hat{S}(\theta_i))^2}{(\hat{S}(\theta_i))^2} \\ \text{s.t.} \quad & \det(S(\phi)) > 0, \quad \phi = \{0^\circ, 1^\circ, 2^\circ, \ldots, 180^\circ\} \end{aligned} \tag{S12}$$

where $\hat{S}(\theta_i)$ represents either the measured data or the FEA modeled data for each independent model fit respectively, Θ represents the set of angles that resolve the data (0° to 180° in 22.5° increments for measured data and 5° increments for FEA data) and the constraint on the determinant is applied to a more fine angular increment ϕ every 1° to the model to ensure the it is physically realistic at all points. The final optimized coefficients for the modeled data are provided in Table S2.

The measured and modeled data are presented in the main text Figure 2. Here we include a twin figure (Figure S3 to show the FEA simulated and FEA fit model. Here we see even better agreement between the FEA data and model as compared to the measured data and its respective model. Notably, the FEA

Table S2: Analytical model fit coefficients for each measured and FEA data set. Reference Equation S8 and Equation S9 for placement of coefficients in S matrices.

Data Set	c_1	c_2	c_3	c_4	c_5	c_6
2*22 Measured	2.606	3.018	2.336	0.072	3.720	4.908
2*22 FEA	3.092	3.150	3.061	-0.003	6.197	6.321
442 Measured	2.868	2.591	-0.120	-2.346	2.753	3.727
442 FEA	2.904	2.826	0.064	-2.855	5.636	5.755

model predicts a higher stiffness than the measured results indicate; which is also borne out in the larger coefficients of Table S2. However, the general trends are all qualitatively well-matched (e.g., 2*22 peaks in moduli or asymptotes in Poisson's ratio; 442 constant Young's moduli sinusoidal normal-shear coupling). This then validates the models derived in Equation S8 and Equation S9 as characteristic of the materials' behavior across the full range of θ and indicates suitability for use against the measured data in the main text.

3 Eigenvalue Normalization and Gamut

To study the transitions in SLM lattices, we introduce the concept of the "extremal material gamut" based on normalized eigenvalues ordered from smallest to largest.

$$\hat{\lambda}_i = \frac{\lambda_i}{\lambda_3}, \quad \text{for } i \in \{1,2,3\} \text{ and } \lambda_1 \leq \lambda_2 \leq \lambda_3 \tag{S13}$$

Normalization of eigenvalues fulfills two functions: it facilitates rapid evaluation of extremal materials by highlighting smaller eigenvalues and permits comparisons across different material symmetries by focusing on extremal attributes instead of direct stiffness.

Furthermore, normalized eigenvalues help define the eigenvalue gamut, a feasible space delineated by $\hat{\lambda}_1 = \hat{\lambda}_2$, $\hat{\lambda}_2 = \hat{\lambda}_3 = 1$, and $\hat{\lambda}_1 = 0$ within the unit square. Isotropic materials — characterized by two equal eigenvalues [1] — are located along the diagonal and the upper edge of this triangle. The left boundary, marking anisotropic limits, exhibits no equal eigenvalues except at the endpoints, but always includes extremal points, with $\hat{\lambda}_1 = 0$ denoting ideal unimodal and bimodal materials at $(0,1)$ and $(0,0)$ respectively, encompassing all intermediate unimodal points.

4 Test and Characterization of Array

Array Construction

A 4×18 square lattice array was fabricated to validate and characterize the 2*22 and 442 materials (Figure S4). The design incorporates snap-fit connectors and a rotational dovetail, facilitating reconfigurable and easy assembly (Figure S5). This modular construction allows for the easy replacement of parts because no parts are rigidly connected, instead relying on friction and other forces to maintain contact. A ratcheting scheme aligns and secures the SLMs during testing (Figure S5b). The close fit of the dovetail seen in the section view Figure S5c enables the SLM to pivot while maintaining its connection during loading for a range of θ.

The dovetail's length determines the array's available configuration space. Dovetails in the samples extend over 135° (Figure S5a), allowing testing within $\theta \in [0, 135°]$. However, this precludes reprogrammability within $\theta \in (135°, 180°)$, where the dovetails contact the snap-fit base, immobilizing the SLM and eliminating all compliance. This restriction serves as a tunable parameter, offering potential areas for future investigation.

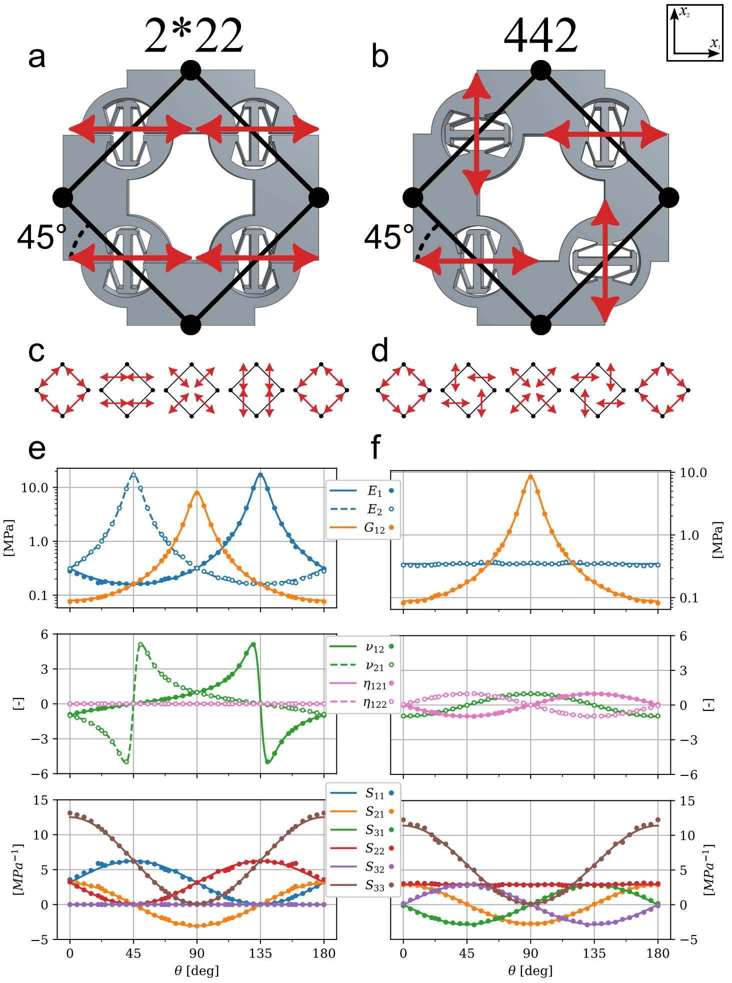

Figure S3: A twin figure to the main text Figure 2, here showing FEA simulated and FEA fit model data. CAD models used for FEA for 2*22 (a) and 442 (b) square symmetry materials shown at a $\theta = 45°$ configuration. The illustrations below each image (c and d) show the DOF alignment for each symmetry as a function of θ and correspond to the major x-axis grid lines in the plots below. The data shown in (e) and (f) show experimental data (dots) and corresponding analytical fits (solid/dashed lines). From top to bottom: the directional Young's and shear moduli (E_1, E_2 and G_{12}); the Poisson's and normal-shear coupling ratios (ν_{12}, ν_{21} and η_{121}, η_{122}); and finally the individual S matrix components which were used to fit the model directly. Compared to the main text Figure 2, interquartile ranges are not shown due to each data point having only one simulation run.

a

b

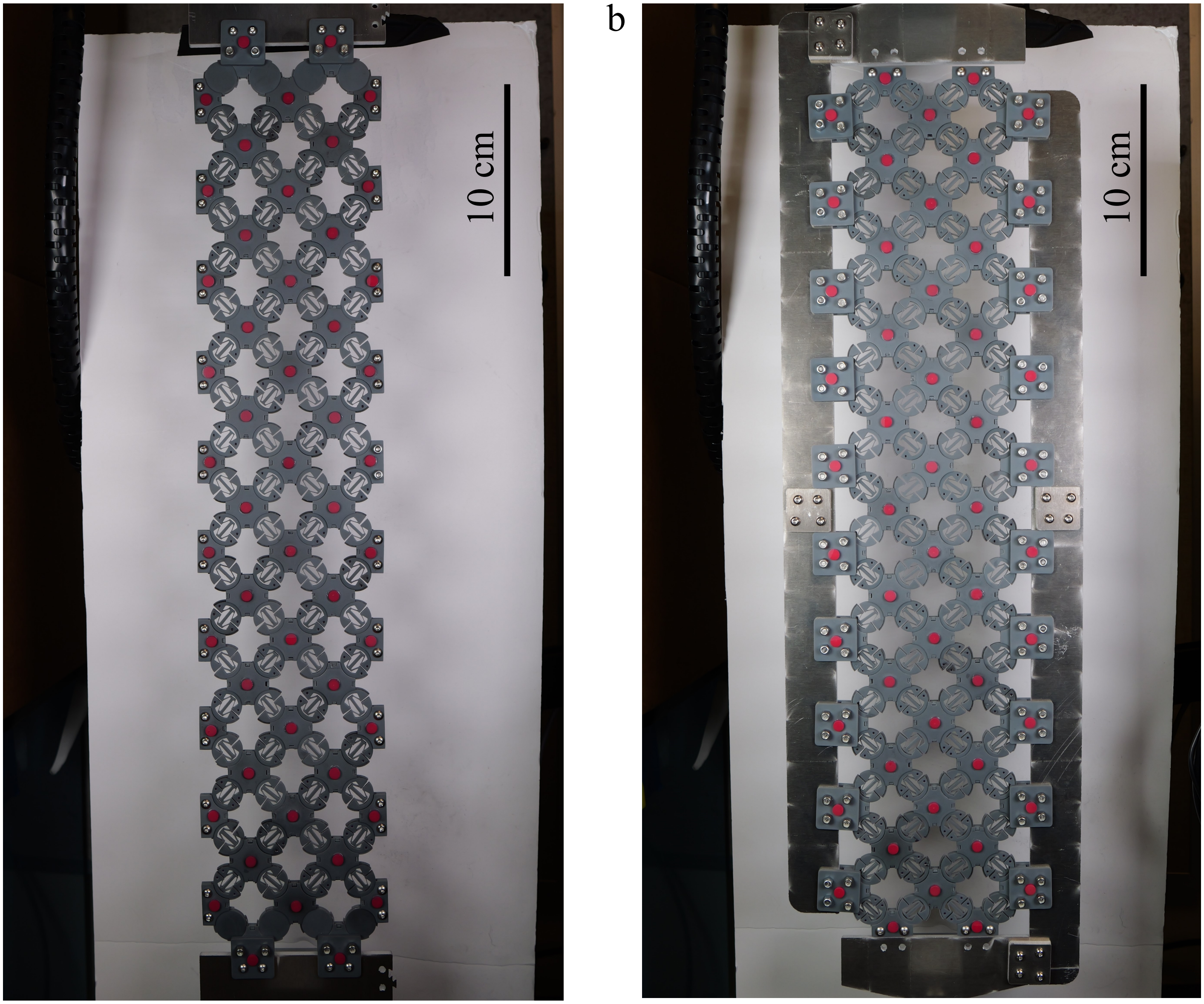

Figure S4: The 4 × 18 array under tensile loading (a) and shear loading (b). Red markers are used for optical tracking to extract local strain information in the array.

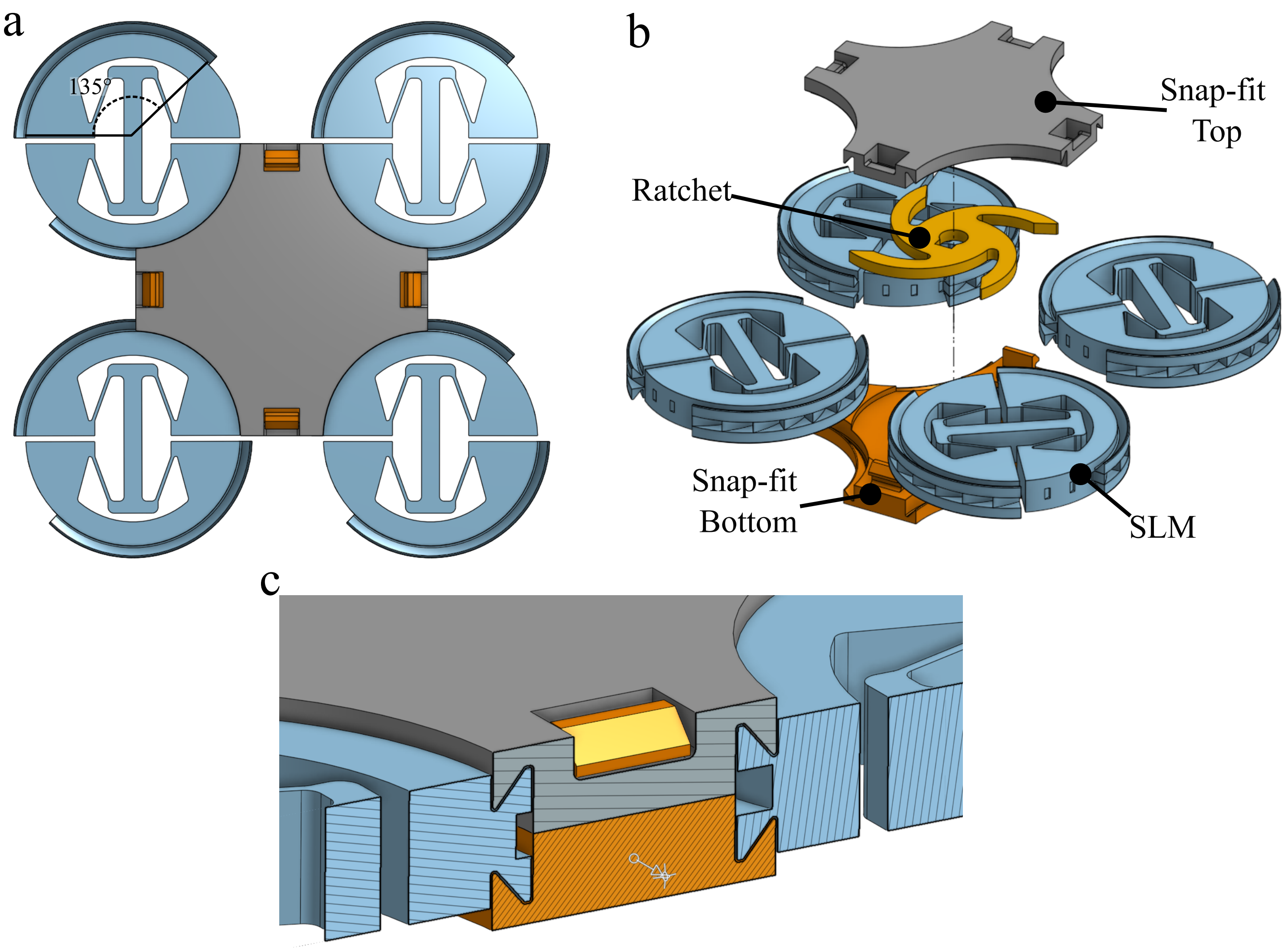

Figure S5: CAD of the SLM reprogrammable array unit cell. SLMs are arranged on a square lattice (a) and attached by running a dovetail through a snap-fit enclosure (b) so that the SLM is captured at least on one side. There is a hidden, internal ratchet spring system to help align and secure the SLMs during testing. (c) shows a close-up section view of the dovetail, illustrating how the SLM is allowed to pass through the enclosure, while maintaining contact.

Test, Measurement, and Characterization

The array in Figure S4 was tested on a universal testing machine (UTM) (Instron 5965) in a quasi-static manner. Two symmetry patterns (2*22 and 442) were tested, in two different loading conditions (tensile and shear), with approximately five tests per θ value for $\theta \in 0, 22.5°, 45°, 67.6°, 90°, 112.5°, 135°$. The force vs. displacement curves and videos of the array deforming array were recorded for testing at various θ configurations. The global force was used for characterization, while the middle third of the optical trackers were used to capture deformation behavior. A subset of the trackers were used to avoid boundary effects at the top and bottom end of the array.

5 Table of Material Values

Material values for the materials seen in Figure 1 from the main document are shown in Table S3 and Table S4.

Media Legends

5.1 Movie M1:

In-plane, orientation dependent material properties of the 2*22 array. As the angle of the Straight Line Mechanisms (SLM) vary relative to the C4 symmetry of the lattice, the materials relative Young's Modulus and shear modulus evolve. The material transitions from positive Poisson Ratio, to zero and ultimately a negative Poisson's ratio material.

5.2 Movie M2:

In-plane, orientation dependent material properties of the 442 array. As the angle of the Straight Line Mechanisms (SLM) vary relative to the C4 symmetry of the lattice, the materials relative Young's Modulus and shear modulus evolve by clocking and changing magnitude.

5.3 Movie M3:

In-Situ array reprogramming. We demonstrate the process of changing the arrays properties without reconstruction of the array.

References

[1] G. W. Milton, A. V. Cherkaev, *J. Eng. Mater. Technol.* **1995**, *117*, 4 483.

[2] T. Frenzel, M. Kadic, M. Wegener, *Science* **2017**, *358*, 6366 1072.

[3] H. Nassar, Y. Y. Chen, G. L. Huang, *J. Mech. Phys. Solids* **2019**, *129* 229.

[4] X. Xu, C. Wang, W. Shou, Z. Du, Y. Chen, B. Li, W. Matusik, N. Hussein, G. Huang, *Phys. Rev. Lett.* **2020**, *124*, 11 114301.

Table S3: Selection of 2*22 Simulated Material Properties

θ	mode	E_1 [MPa]	E_2 [MPa]	G [MPa]	ν_{12}	η	λ_1 [MPa]	λ_2 [MPa]	λ_3 [MPa]	$\hat{\lambda}_1$	$\hat{\lambda}_2$	$\hat{\lambda}_3$
0°	m=2	0.280	0.310	0.076	-0.909	0.001	0.150	0.153	6.740	0.022	0.023	1.000
45°	m=2	0.162	17.099	0.160	0.001	-0.001	0.162	0.320	17.100	0.009	0.019	1.000
90°	m=1	0.317	0.317	7.880	0.980	0.000	0.160	15.760	16.040	0.010	0.983	1.000

Table S4: 632 Simulated Material Properties

θ	mode	E [MPa]	G [MPa]	K [MPa]	K/G	ν	λ_1 [MPa]	λ_2 [MPa]	λ_3 [MPa]	$\hat{\lambda}_1$	$\hat{\lambda}_2$	$\hat{\lambda}_3$
90°	m=0	16.000	6.040	12.000	1.987	0.330	12.030	12.080	24.070	0.500	0.502	1.000
0°	m=1	0.526	2.190	0.140	0.064	-0.880	0.280	4.378	4.382	0.064	0.999	1.000
90°	m=2	0.394	0.102	6.680	65.490	0.970	0.200	0.204	13.360	0.015	0.015	1.000
0°	m=3	0.131	0.097	0.050	0.513	-0.320	0.099	0.193	0.195	0.510	0.993	1.000
